# Genetic Polymorphisms of Cytotoxic T-Lymphocyte Antigen 4 in Primary Biliary Cholangitis: A Meta-Analysis

**DOI:** 10.1155/2017/5295164

**Published:** 2017-05-31

**Authors:** Xing-Chen Yang, Masayuki Fujino, Song-Jie Cai, Shao-Wei Li, Chi Liu, Xiao-Kang Li

**Affiliations:** ^1^Department of Pharmacy, Longhua Hospital, Shanghai University of Traditional Chinese Medicine, Shanghai, China; ^2^Division of Transplantation Immunology, National Institute for Child Health and Development, Tokyo, Japan; ^3^AIDS Research Center, National Institute of Infectious Diseases, Tokyo, Japan

## Abstract

**Background and Aim:**

The connection between gene polymorphisms of cytotoxic T-lymphocyte-associated protein 4 (*CTLA4*) and primary biliary cholangitis (PBC) is still vague and blurred. The purpose of this study is to precisely estimate the association of the polymorphisms of *CTLA4* with the risk of PBC by using a meta-analysis.

**Methods:**

PubMed and the Chinese National Knowledge Infrastructure (CNKI) database were used to search correlative literatures, and the documents which were about the relationships between the polymorphisms of *CTLA4* (rs231775, rs231725, rs3087243, and rs5742909) and PBC were collected as of June 2016. The strength of correlation based on odds ratios (ORs) and its 95% confidence intervals (95%CIs) was computed by STATA.

**Results:**

Generally, in rs231775, a significant risk was found in G allele, the value of OR was 1.32, and its 95%CI was 1.19 to 1.47. The same situation was found in A allele of rs231725, the value of OR was 1.33, and its 95%CI was 1.22 to 1.45. As genotypic level, different genotypic models were also found to have obvious relevance with PBC in rs231775 and rs231725. No obvious connections were found in other SNPs.

**Conclusion:**

This study indicated that the polymorphisms of rs231775 and rs231725 would be the risk factors of PBC.

## 1. Introduction

Autoimmune diseases are resulted from the dysfunction of the immune system, which generate immune response to autoantigens. Primary biliary cholangitis (PBC) is one kind of specific autoimmune diseases, which can cause progression of fibrosis and cirrhosis in the liver and lead to liver failure finally [1–4]. There are still no special treatments for primary biliary cholangitis in the world. At present, the most efficient therapy is to use ursodeoxycholic acid for those who are in early period of primary biliary cholangitis. However, ursodeoxycholic acid could not still stop the progression of the disease. When the final stage of PBC occurred, the only therapy is liver transplantation. Up to now, the definite etiology of PBC is still not clear.

Cytotoxic T-lymphocyte-associated protein 4 (CTLA4) is expressed on the surface of activated T and transmits inhibitory signal, and it is also found on the surface of regulatory T cells. The functions of CTLA4 are to lower responses of T cell and maintain peripheral tolerance of T cell [5]. The abnormal costimulation between specific autoreactive T lymphocytes and CTLA4 in PBC patients causes the reaction of peripheral T lymphocyte not to be terminated, which might be one of the pathogens of PBC.

As to PBC, CD8^+^ T cells are important factors in the pathogenesis [6]. CD8^+^ T cells are sensitive to E2 components of pyruvate dehydrogenase complexes (PDC-E2) which are abnormally expressed on the surface of biliary epithelial cells (BECs) and would result in apoptosis for these epithelial cells and destruction of the small bile duct [7, 8]. As a coinhibitor signal, CTLA4 binds to CD80/CD86 on antigen-presenting cells (APCs) with higher affinity comparing with CD28 [9]. Binding with CD80/CD86 to deliver negative signal into T cells, CTLA4 can result T cell responses in inhibition or termination [10]. Then, CTLA4 can regulate immune suppression and peripheral tolerance in CD8^+^ T cells. Thus, CTLA4 could be involved in the regulation of pathological processes of PBC, which might be a therapeutic for PBC. Two studies demonstrated that treatment with CTLA4-Ig, which can reduce self-reactive T cell activation and liver inflammation significantly, could obviously reduce the level of portal inflammation and biliary cell damage in the mouse model [11, 12].

So, CTLA4 regulation plays an important role in the pathological process of PBC. Meanwhile, the results, which were shown in some researches about the treatment of *CTLA4*, have shown that *CTLA4* plays a unique role in the pathogenesis and treatment of PBC.

Recently, genetic factors are deemed to be an important role in PBC, which is mainly in favor of familial clustering of PBC [13]. Recent study showed that PBC was significantly associated with some concrete gene polymorphisms [14]. Since PBC displays characteristics of autoimmunity, more and more studies concentrated in associations between genetic polymorphism and variations of autoimmunity.

There are several evidences to prove the connections between polymorphisms of *CTLA4* and other autoimmune diseases in recent literatures [15–17]. In recent years, there are extensive researches about the links between *CTLA4* and PBC. rs231775, rs231775, rs3087243, and rs5742909 are the most common four single-nucleotide polymorphisms (SNPs) to be widely studied [18–22]. Thus, with changes of the function in these SNPs, the possibility of PBC might be increased. Because of inconclusive connections between the polymorphisms of *CTLA4* and the risks of PBC, those relative researches are necessary to be combined to conduct a meta-analysis. Several early systematic reviews which had been published mainly regarded the relationships between the polymorphisms in several SNPs and primary biliary cholangitis [23–25]. However, these studies either did not draw the clear conclusion or did not include some latest literatures. So, in this research, 16 studies are combined to analyze the correlation between the polymorphisms of *CTLA4* and risks of PBC [1, 18–22, 26–35].

## 2. Materials and Methods

### 2.1. Study Selection and Data Extraction

We used PubMed and the China Knowledge Resource Integrated database up to June 2016, and related literatures about the relationships between the polymorphisms of *CTLA4* and risks of PBC were researched on computer with retrieval words (“primary biliary cholangitis, PBC, cytotoxic T-lymphocyte antigen 4, Polymorphism, SNP, genetic variants”). At last, we found 26 studies contained relative contents about *CTLA4* and PBC.

The following conditions should be met in studies: firstly, literature should be a case-control study; secondly, outcome was about primary biliary cholangitis; and thirdly, odds ratio and its 95% confidence interval should be estimated with adequate data in literatures. Exclusive criteria: insufficient information for data extraction. At last, this meta-analysis included 16 literatures after 10 literatures were excluded.

According to the inclusion conditions mentioned above, two researchers extracted data independently. The researchers gathered these data from each study: SNPs, name of the first author, date of publication, ethnicity, number of allele, and genotype. Diversities among researchers were solved with discussion.

## 3. Statistical Analysis

The intensity of associations between PBC risk and *CTLA4* polymorphisms was assessed in a random-effect model or fixed-effect model by the estimated OR and its 95%CI. Assessing the difference between the *CTLA4* polymorphisms and the PBC risk in Caucasian and Asian was also conducted by using subgroup analyzing. *Z*-test was used to compute the significant difference of pooled OR. The *p* value of *Z*-test was calculated to access significance. Because multiple comparisons were conducted in this study, the threshold of *p* values was corrected with formula 1 − (1 − *p*)^1/n^ for the Bonferroni correction [36]. *Q*-test was used to assess heterogeneity and calculate *I*^2^ statistic. When *p* was less than 0.05 or *I*^2^ was more than 50%, the results among the studies indicated significant heterogeneity. In addition, possible publication biases were estimated by Begg's funnel plot and Egger's regression; *p* value was calculated to access bias. When *p* was less than 0.05, a publication bias was considered to be existence. The validity and reliability of a meta-analysis were evaluated by conducting sensitivity analysis [37]. All the statistics were performed by STATA 14 software.

In this study, linkage disequilibrium (LD) was used to measure the association among these four *CTLA4* SNPs after multiple comparison. The values of *D*' and *r*^2^ were calculated by SHEsis software (http://analysis.bio-x.cn/myAnalysis.php) [38]. When *D*' > 0.8 or *r*^2^ > 0.4, linkage disequilibrium could be considered.

## 4. Results

### 4.1. Literature Search

In total, the number of cases and controls were 4422 and 5210 in 16 studies, respectively (Table 1). As to *CTLA4*, the SNPs which were mostly consulted were rs231775, rs231725, rs3087243, and rs5742909. These SNPs were reported in 14, 6, 9, and 5 studies, respectively. The genotypes of controls were in line with Hardy-Weinberg equilibrium in most articles.

### 4.2. Meta-Analysis

Obvious heterogeneity was identified in *CTLA4* rs231775 polymorphism (G versus A: P(het) = 0.008, *I*^2^ = 54.1%; GG versus AA: P(het) = 0.032, *I*^2^ = 45.6%), rs3087243 polymorphism (GA versus GG: P(het) = 0.028, *I*^2^ = 53.5%; AA + GA versus GG: P(het) = 0.037, *I*^2^ = 51.2%), and rs5742909 polymorphism (T versus C: P(het) = 0.001, *I*^2^ = 69.4%; TC versus CC: P(het) = 0.01, *I*^2^ = 66.7%; and (TT + TC) versus CC: P(het) = 0.007, *I*^2^ = 69%). Therefore, we chose the random-effect model to generate extensive CIs in these genetic models, the rest of genetic models were used the fixed-effects model (Table 2).

The results of this analysis in the association of *CTLA4* polymorphisms (rs231775, rs231725, rs3087243, and rs5742909) with susceptibility to PBC are presented (Table 3).

**Figure 1:**
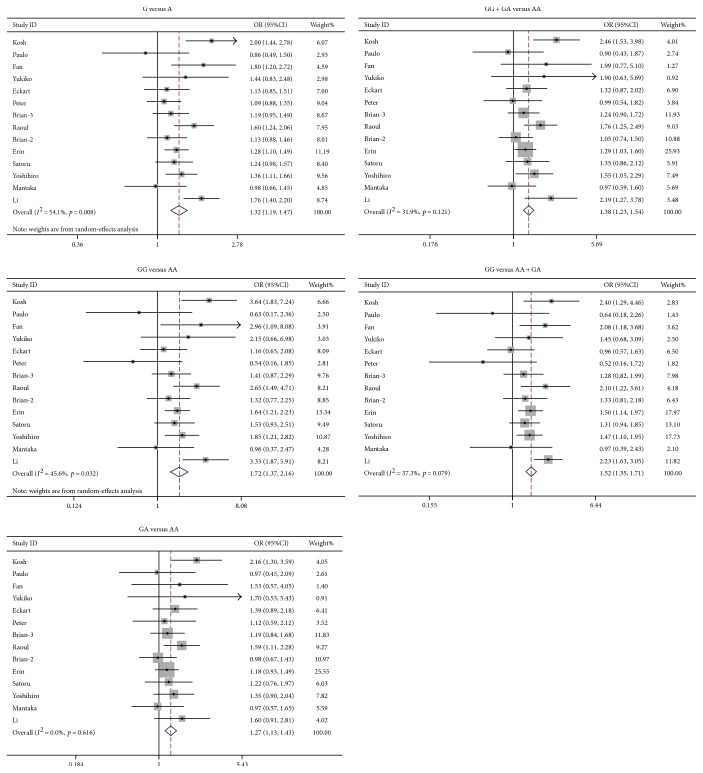
Odds ratio with its 95% confidence interval of PBC linked with *CTLA4* rs231775. The rhombus represented the pooled OR with 95%CI.

**Figure 2:**
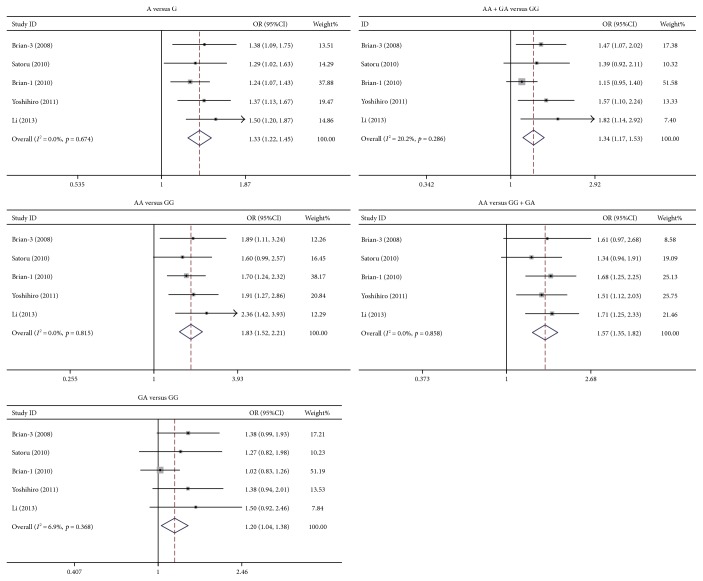
Odds ratio with its 95% confidence interval of PBC linked with *CTLA4* rs231725. The rhombus represented the pooled OR with 95%CI.

**Figure 3:**
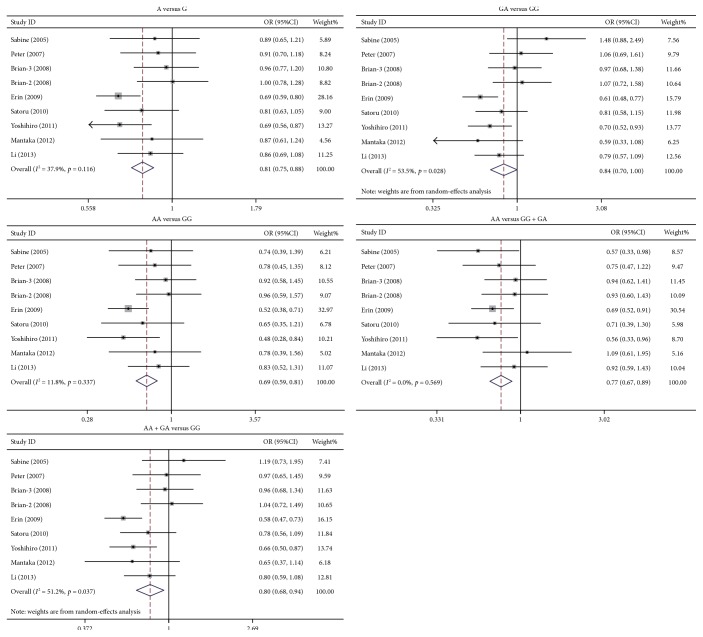
Odds ratio with its 95% confidence interval of PBC linked with *CTLA4* rs3087243. The rhombus represented the pooled OR with 95%CI.

**Figure 4:**
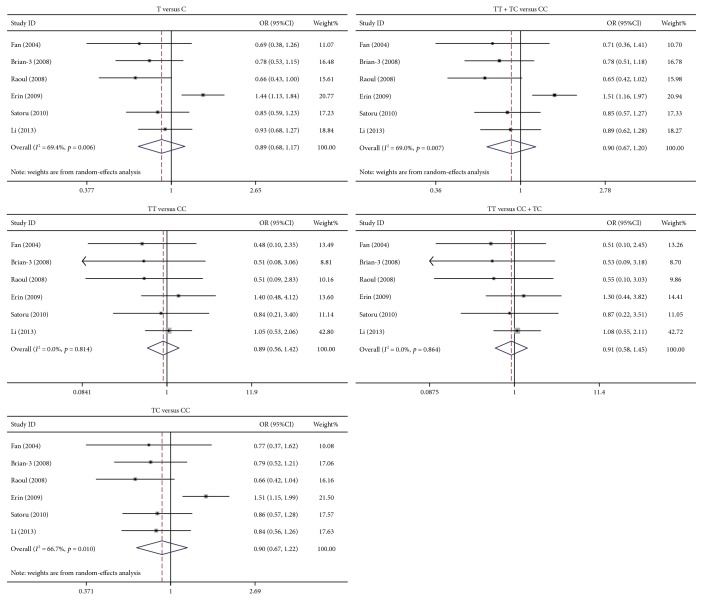
Odds ratio with its 95% confidence interval of PBC linked with *CTLA4* rs5742909. The rhombus represented the pooled OR with 95%CI.

The study identified that rs231775 polymorphism of *CTLA4* was significantly associated with PBC susceptibility. The ORs (95%CIs) of G versus A, GG versus AA, GA versus AA, (GG + GA) versus AA, and GG versus (AA + GA) were 1.32 (1.19–1.47), 1.72 (1.37–2.16), 1.27 (1.13–1.43), 1.38 (1.23–1.54), and 1.52 (1.35–1.71), respectively. As to each model, the *p* value was below 0.0001 (Figure 1). The rs231725 polymorphism also showed significant association with PBC susceptibility. The ORs with 95%CIs of A versus G, AA versus GG, GA versus GG, (AA + GA) versus GG, and AA versus (GG + GA) were 1.33 (1.22–1.45), 1.83 (1.52–2.21), 1.20 (1.04–1.38), 1.34 (1.17–1.53), and 1.57 (1.35–1.82), respectively. As to each genetic model, the *p* value was below 0.05 (Figure 2). Nevertheless, no association was identified between rs3087243 (Figure 3) and rs5742909 (Figure 4) polymorphisms and PBC susceptibility. Subgroup analysis showed that both rs231775 and rs231725 showed significant association with PBC susceptibility for Asians and for Caucasians.

### 4.3. Calculation of Linkage Disequilibrium

Based on the values of *r*^2^, there was no obviously linkage disequilibrium among four SNPs (Table 4).

### 4.4. Sensitivity Analyses and Publication Bias

**Figure 5:**
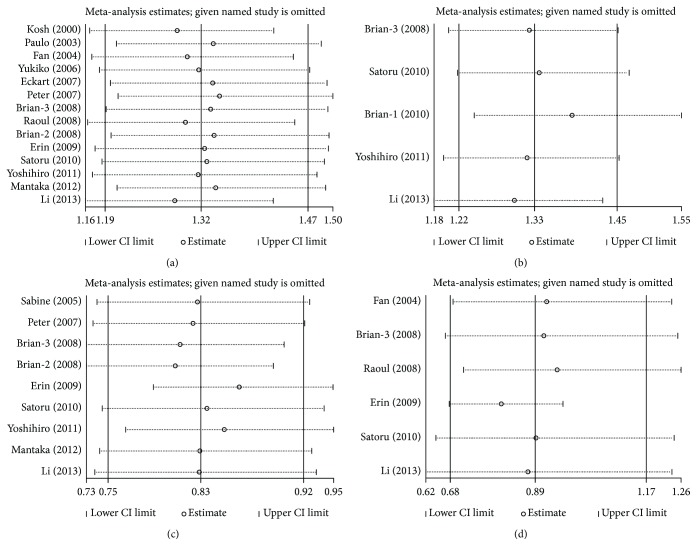
Sensitivity analysis for the effect of *CTLA4* and PBC: (a) rs231755 (G versus A), (b) rs231725 (A versus G), (c) rs3087243 (A versus G), and (d) rs5742909 (T versus C).

**Figure 6:**
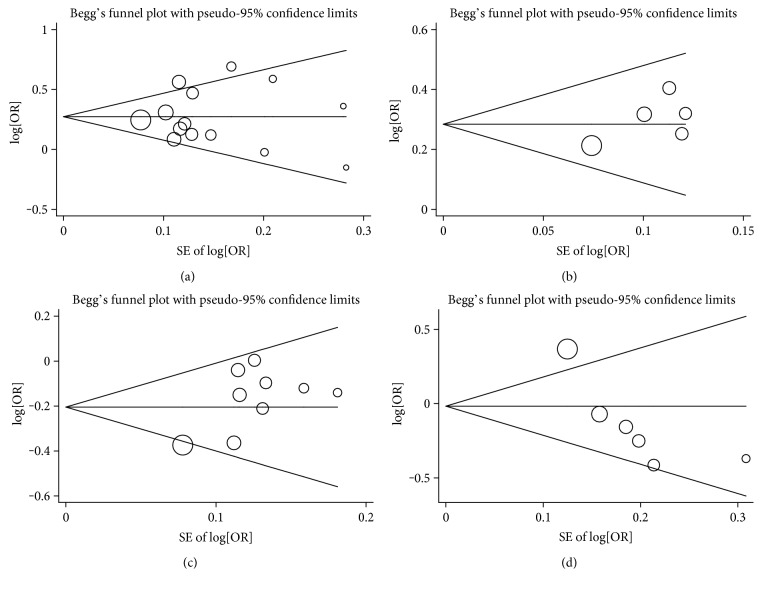
Publication bias for the influence of *CTLA4* and PBC: (a) rs231755 (G versus A), (b) rs231725 (A versus G), (c) rs3087243 (A versus G), and (d) rs5742909 (T versus C).

Sensitivity analysis was conducted by omitting each studies sequentially, suggesting that the results for the overall population were statistically stable and reliable (Figure 5). Publication bias was examined by using Egger's regression and Begg's funnel plot in our research. No obvious publication bias was identified. For Begg's test, *p* values of rs231775 (G versus A), rs231725 (A versus G), rs3087243 (A versus G), and rs5742909 (T versus C) were 0.661, 0.806, 0.466, and 0.060, respectively. For Egger's test, *p* values of genetic models mentioned above were 0.952, 0.186, 0.061, and 0.029, respectively (Figure 6).

## 5. Discussion

Multiple comparisons were conducted in this study. To minimize the type I error, the threshold of *p* values was corrected by the Bonferroni correction. The Bonferroni correction compensates for that increase by testing each individual hypothesis at a significance level [36]. Then, the threshold of *p* value for the Bonferroni correction can be calculated with the corresponding critical values 1 − (1 − *p*)^1/*n*^. There are four SNPs in this study for multiple comparisons, and the original threshold of *p* value was 0.05. So, after calculating with above formula (*p* = 0.05, *n* = 4), *p* < 0.0127 was considered statistically significant.

As to the polymorphisms of rs231775 and rs231725, significant connections were found to be associated with PBC in all 5 genetic models. For patients in cases, the frequencies of allele and genotype in rs231775 and rs231725 were increased more significantly than those in controls. As to allele, the results of rs231775 were similar to the results of five published meta-analyses by Eskandari-Nasab et al. [39], Huang et al. [23], Miyake et al. [24], Li et al. [25], and Chen et al. [14]. Li and Miyake indicated that the G allele might be connected with PBC as a risk factor. On the contrary, meta-analyses from Chen and Huang proposed that the relationship between G allele and susceptibility of PBC was observed only in Asian.

As to rs3087243, our analysis showed that both allele and genotype were negative associations with PBC in overall populations. For rs5742909, in codominant, dominant, and recessive models, there were no connections with susceptibility of PBC in Caucasian and Asian. These results were consistent with those in one meta-analysis, which was conducted by Li et al. [25], including 12 studies.

Through subgroup analysis, GG homozygosity of rs231775 and AA homozygosity of rs231725 were associated with the susceptibility to PBC both in Asians and in Caucasians. AA homozygosity of rs3087243 was protective against PBC in Asians and Caucasians. On the other hand, GA heterozygosity of rs231725 was associated with the susceptibility to PBC in Asians although it was not in Caucasians. GA heterozygosity of rs3087243 was protective against PBC in Asians although it was not in Caucasians. Thus, there may be a little different between Asians and Caucasians in the relationship between SNP polymorphism of *CTLA4* and the susceptibility to PBC. In order to solve these problems, further studies in various ethnicities are required.

In linkage disequilibrium, coefficients *D*' and *r*^2^ were frequently used. They have quite different characteristics and could be applied for different purposes. Typically, *r*^2^ is useful in the context of association studies, *D*' is the measure of choice to assess recombination patterns in a given population [40]. It is indicated that the two loci were not recombined and were in a complete linkage disequilibrium when the value of *D*' is 1. But the significance of values would be difficult to interpret when *D*' < 1. Meanwhile, when the sample size is small and the frequency of SNPs is low, the estimate of *D*' would be too large. In this case, even the sites of linkage equilibrium can get larger *D*' value, the actual meaning of the *D*' could easily be exaggerated. Then, the value of *r*^2^ could be more reliable under this condition. In this study, the frequency of genotypes in rs5742909 was much lower than the others (Table 1). Thus, the values of *D*' were much larger than the value of *r*^2^. So, *r*^2^ was chosen to assess the linkage disequilibrium. Based on the results, LD was not observed among four SNPs.

Heterogeneity may affect pooled results as one of possible factors. It can be categorized into heterogeneity of the genetic model and effect. In this study, a relatively moderate heterogeneity was heeded. Among 16 studies, HWE values of five studies were out. As to rs231775 and rs5742909, there were three and two studies to be out of HWE, respectively. Thus, we conducted the sensitivity analysis in all studies. In the analysis of rs231775, the results of *I*^2^values reduced when we removed the article by Li et al. [22]. The heterogeneity in Caucasians was larger than in Asians. This study showed that diversity of genetic ethnicities or methodological differences might be the sources of heterogeneity.

There are some characteristics in this meta-analysis. Comparing with other similar articles, we conducted four SNPs in one study, and each SNP included five different genetic models. We assessed subgroup by analyzing ethnicities and obtained more precise estimation of the relationships. We also performed sensitivity analysis to test the validity of the results.

To date, several genome-wide association studies (GWAS) and genome-wide meta-analysis on PBC have been performed. From these literatures [41–47], there were several genes to be identified as significant susceptibility loci for PBC. There were ethnic differences in genetic susceptibility loci such as *TNFSF15*, *POU2AF1*, *IL12A*, and *IL12RB2* and common pathogenic pathways such as B cell differentiation, IL-12 signaling, and T cell activation.

As to GWAS, the relationship between mutations of SNPs and occurrence of disease might not be a necessity but a probability. So, a large number of samples should be analyzed for the association study between gene and disease. The number of cases which were enrolled in each publication of genome-wide association study of PBC was not enough comparing with the probability of gene mutation. Thus, some higher risk loci with lower mutation frequency could be concealed by lower risk loci with higher mutation frequency. Meanwhile, it was different from the GWAS that focused on the onset of disease, and the data of our meta-analysis might provide a point in the search for novel therapies that are urgently needed to improve outcomes for PBC patients.

On the one hand, GWAS efforts have focused on the identification of association of genetic variants with PBC, but not with specific properties of disease such as response of treatment [48]. As mentioned, the IL-12 pathway has been strongly implicated in the pathogenesis of PBC. The monoclonal antibody took the IL-12p40 subunit as the target and exerted its effect on both the IL-12/TH1 and IL-23/TH17 axes. While the monoclonal antibody has demonstrated therapeutic benefit in patients with Crohn's disease and psoriasis, none of the patients achieved the predefined primary endpoint of alkaline phosphatase reduction from baseline [10, 49]. Although, the data of *CTLA4* polymorphism and the association between *CTLA4* and PBC were not reported in the GWAS of PBC, *CTLA4* was the main focus of PBC in many candidate gene studies, and certain benefit results were obtained as a therapeutic target from *CTLA4*. CTLA4-Ig has been developed as an exciting outcome in mouse model of PBC [11, 12]. Based on these studies, a new clinical study has been set to determine the effect of abatacept in PBC patients who have no response to UDCA (NCT02078882).

On the other hand, IL-12 signal pathway may be an important role for PBC through Th1/2 differentiation among these loci from GWAS, but *CTLA4* could also impact Th1/2. Indeed, *CTLA4*-deficient mice and T cells were shown to be strongly trend a Th2 phenotype [50]. This is the control of Th1/Th2 differentiation, which was shown to depend both on the cytokine microenvironment and costimulatory signals [51]. It is evidenced that some gene loci could be potential risk for PBC in GWAS, but these findings still have not translated into clinic. Although, the polymorphism of *CTLA4* could not be improved in GWAS, as to biliary cell damages in PBC, *CTLA4* could influence the effect of these inflammatory cytokines, such as IL-12, and IL-23 [52].

In view of the publication of the GWAS of PBC, our meta-analysis might be quite basic. However, we have collected sufficient documentations that have ever been published and analyzed four SNPs of *CTLA4* that have ever been reported in the publication of candidate gene studies. It should be said that this study was a more comprehensive meta-analysis of association between *CTLA4* and PBC. Our findings might illustrate that relevant research could still be gained from the candidate gene investigation.

In this meta-analysis, there were still some limitations to be existed. First, except race, there were other factors to be concerned, which included age, gender, and alcohol habit. It would be useful to understand that different risk factors might interact with the development of PBC as genetic variations. Secondly, some studies suggested that patients in PBC would also suffer other autoimmune diseases. Since, these literatures which we included did not mention whether other diseases were excluded in those patients, these situations may introduce errors during analyzing.

To sum up, this meta-analysis showed that the GG, GA genotype, and G allele of rs231775 and AA, GA genotype, and A allele of rs231725 in *CTLA4* may be risk factors for PBC in Asians and Caucasians. AA, GA genotype, and A allele of rs3087243 may be negatively associated with PBC in overall populations, especially in Asian. There was no significant connection with PBC in rs5742909 of *CTLA4*. Not only the impact on cytokine regulation but also the benefit results as therapeutic target, to some extent, *CTLA4* still plays a role which could not be completely ignored in PBC.

## Figures and Tables

**Table 1:** Basic characteristics of involved studies.

SNP	First author	Year	Race	Case					Control					P(HWE)
				A	B	AA	AB	BB	A	B	AA	AB	BB	
rs231775	Kosh	2000	C	131	115	35	61	27	278	122	99	80	21	0.613
	Paulo	2003	C	69	31	23	23	4	88	46	29	30	8	0.955
	Fan	2004	A	46	108	6	37	34	139	181	23	93	44	0.021
	Yukiko	2006	A	30	60	5	20	20	61	85	14	33	26	0.545
	Eckart	2007	C	206	154	58	90	32	243	161	78	87	37	0.149
	Peter	2007	C	389	243	40	29	4	497	285	54	35	10	0.716
	Brian-3	2008	C	414	288	122	170	59	352	206	111	130	38	0.995
	Raoul	2008	C	313	203	95	123	40	407	165	145	118	23	0.883
	Brian-2	2008	C	421	281	131	161	59	258	152	79	99	27	0.644
	Erin	2009	C	545	417	162	223	96	1562	934	493	577	178	0.661
	Satoru	2010	A	226	390	42	143	123	224	312	47	131	90	0.955
	Yoshihiro	2011	A	314	586	55	204	191	313	429	66	181	124	0.997
	Mantaka	2012	C	144	56	52	40	8	226	90	81	64	13	<0.001
	Li	2013	A	180	444	20	140	152	312	438	49	214	112	0.001

rs231725	Brian-3	2008	C	442	260	139	164	48	391	167	137	117	25	0.998
	Satoru	2010	A	250	368	51	149	108	250	286	58	133	77	0.968
	Brian-1	2010	C	1091	641	368	357	141	1032	490	350	332	79	0.984
	Yoshihiro	2011	A	351	549	68	214	168	347	395	81	185	105	0.977
	Li	2013	A	204	420	29	146	137	316	434	59	198	118	0.109

rs3087243	Sabine	2005	C	167	141	40	87	27	170	162	49	72	45	0.089
	Peter	2007	C	222	168	59	104	32	301	251	82	137	57	0.987
	Brian-3	2008	C	407	295	118	171	62	318	240	91	136	52	0.925
	Brian-2	2008	C	400	302	117	168	66	234	176	70	94	41	0.358
	Erin	2009	C	602	360	198	205	78	1335	1161	362	613	273	0.656
	Satoru	2010	A	454	162	167	120	21	372	164	129	114	25	0.979
	Yoshihiro	2011	A	689	211	264	161	25	515	227	179	157	35	0.946
	Mantaka	2012	C	107	93	32	43	25	158	158	37	84	37	0.426
	Li	2013	A	430	194	159	112	41	492	258	170	152	53	0.048

rs5742909	Fan	2004	A	138	16	63	12	2	274	46	122	30	8	0.003
	Brian-3	2008	C	646	56	297	52	2	502	56	226	50	3	0.9
	Raoul	2008	C	477	39	220	36	2	509	63	226	56	4	0.803
	Erin	2009	C	852	110	377	99	5	2291	205	1055	183	10	0.509
	Satoru	2010	A	550	66	245	59	4	470	66	206	58	4	0.971
	Li	2013	A	541	83	246	49	17	644	106	288	68	19	<0.001

C: Caucasian; A: Asian; P(HWE): *p* value of Hardy-Weinberg equilibrium for control.

**Table 2:** The results of heterogeneity.

SNP	Genetic model	*I* ^2^ (%)	P(het)	Effect model
rs231775	G : A	54.1	0.008	Random
	GG : AA	45.6	0.032	Random
	GA : AA	0.0	0.616	Fixed
	(GG + GA) : AA	31.9	0.121	Fixed
	GG : (AA + GA)	37.3	0.079	Fixed

rs231725	A : G	0.0	0.674	Fixed
	AA : GG	0.0	0.815	Fixed
	GA : GG	6.9	0.368	Fixed
	(AA + GA) : GG	20.2	0.286	Fixed
	AA : (GG + GA)	0.0	0.858	Fixed

rs3087243	A : G	37.9	0.116	Fixed
	AA : GG	11.8	0.337	Fixed
	GA : GG	53.5	0.028	Random
	(AA + GA) : GG	51.2	0.037	Random
	AA : (GG + GA)	0.0	0.569	Fixed

rs5742909	T : C	69.4	0.001	Random
	TT : CC	0.0	0.814	Fixed
	TC : CC	66.7	0.01	Random
	(TT + TC) : CC	69.0	0.007	Random
	TT : (CC + TC)	0.0	0.864	Fixed

P(het): *p* value of *Q*-test for heterogeneity test; *I*^2^: the proportion of total variation contributed among study variants; P(het) < 0.05 or *I*^2^ > 50% indicated significant heterogeneity, using a random model; otherwise, using a fixed model.

**Table 3:** Results of *CTLA4* polymorphisms and PBC.

SNP	Genetic model	Asian			Caucasian			Overall		
		OR	95%CI	*p*	OR	95%CI	*p*	OR	95%CI	*p*
rs231775	G : A	1.47	1.27, 1.72	<0.0001	1.24	1.09, 1.42	0.001	1.32	1.19, 1.47	<0.0001
	GG : AA	2.10	1.55, 2.84	<0.0001	1.51	1.12, 2.04	0.006	1.72	1.37, 2.16	<0.0001
	GA : AA	1.39	1.08, 1.79	0.011	1.24	1.09, 1.42	0.001	1.27	1.13, 1.43	<0.0001
	(GG + GA) : AA	1.65	1.30, 2.10	<0.0001	1.31	1.16, 1.49	<0.0001	1.38	1.23, 1.54	<0.0001
	GG : (AA + GA)	1.66	1.40, 1.96	<0.0001	1.39	1.18, 1.65	<0.0001	1.52	1.35, 1.71	<0.0001

rs231725	A : G	1.39	1.22, 1.57	<0.0001	1.27	1.12, 1.44	<0.0001	1.33	1.22, 1.45	<0.0001
	AA : GG	1.92	1.47, 2.49	<0.0001	1.75	1.33, 2.29	<0.0001	1.83	1.52, 2.21	<0.0001
	GA : GG	1.38	1.07, 1.76	0.012	1.11	0.93, 1.33	0.236	1.20	1.04, 1.38	0.015
	(AA + GA) : GG	1.57	1.24, 1.99	<0.0001	1.23	1.04, 1.46	0.014	1.34	1.17, 1.53	<0.0001
	AA: (GG + GA)	1.52	1.27, 1.83	<0.0001	1.66	1.29, 2.14	<0.0001	1.57	1.35, 1.82	<0.0001

rs3087243	A : G	0.78	0.68, 0.89	<0.0001	0.83	0.76, 0.91	<0.0001	0.81	0.75, 0.88	<0.0001
	AA : GG	0.66	0.49, 0.90	0.008	0.70	0.58, 0.85	<0.0001	0.69	0.59, 0.81	<0.0001
	GA : GG	0.76	0.63, 0.91	0.003	0.90	0.68, 1.21	0.491	0.84	0.70, 1.00	0.051
	(AA + GA) : GG	0.74	0.62, 0.88	0.001	0.86	0.66, 1.11	0.250	0.80	0.68, 0.94	0.008
	AA : (GG + GA)	0.74	0.55, 1.00	0.050	0.78	0.66, 0.92	0.004	0.77	0.67, 0.89	0.001

rs5742909	T : C	0.87	0.70, 1.08	0.206	0.92	0.55, 1.55	0.766	0.89	0.68, 1.17	0.417
	TT : CC	0.90	0.51, 1.58	0.715	0.88	0.39, 1.99	0.762	0.89	0.56, 1.42	0.636
	TC : CC	0.84	0.64, 1.10	0.199	0.95	0.55, 1.64	0.846	0.90	0.67, 1.22	0.506
	(TT + TC) : CC	0.85	0.66, 1.09	0.200	0.93	0.53, 1.63	0.809	0.90	0.67, 1.20	0.463
	TT : (CC + TC)	0.93	0.53, 1.63	0.804	0.87	0.39, 1.97	0.743	0.91	0.58, 1.45	0.695

OR: odd ratio; 95%CI: 95% confidence interval; *p*: *p* value of *Z*-test for significance test of OR; using the Bonferroni correction, *p* < 0.0127 means statistically significant.

**Table 4:** Results of linkage disequilibrium.

	rs231775	rs231725	rs3087243	rs5742909
rs231775	—	0.908	0.964	0.942
rs231725	0.030	—	0.924	0.921
rs3087243	0.017	0.031	—	0.914
rs5742909	0.020	0.074	0.030	—

Lower left areas are values of *r*^2^. Upper right areas are values of *D*'.
